# High-resolution modified look-locker inversion recovery (HR-MOLLI) for RV extracellular volume fraction at 3T and 1.5T: A feasibility study

**DOI:** 10.1186/1532-429X-16-S1-P159

**Published:** 2014-01-16

**Authors:** Edouard Semaan, Bruce S Spottiswoode, Benjamin Freed, Zoran Stankovic, Bradley D Allen, Maria Carr, Marie Wasielewski, Karissa Campione, Sanjiv Shah, James C Carr, Michael Markl, Jeremy D Collins

**Affiliations:** 1 Department of Radiology, Northwestern University, Chicago, Illinois, USA; 2 Cardiovascular MR R&D, Siemens Healthcare USA, Chicago, Illinois, USA; 3 Department of Cardiology, Northwestern University, Chicago, Illinois, USA; 4 Department of Biomedical Engineering, Northwestern University, Chicago, Illinois, USA

## Background

Cardiac MR (CMR) is the reference standard for assessing macroscopic myocardial scar. Determination of the extracellular volume fraction (ECV) by T1 estimation using the modified Look-Locker inversion recovery (MOLLI) correction enables quantitation of diffuse fibrosis. The purpose of this study is to evaluate the feasibility and reproducibility of an optimized high-resolution MOLLI (HR-MOLLI) technique at 3T and 1.5T for RV ECV calculation in healthy volunteers.

## Methods

25 healthy volunteers (16 males, 41 ± 14.3 yrs) were scanned at 3T (MAGNETOM Skyra, Siemens AG, Healthcare Sector, Erlangen, Germany) and 15 (11 males, 46.4 ± 13.5 yrs) were scanned at 1.5T (MAGNETOM Aera). T1 mapping was performed in the axial orientation using an investigational HR-MOLLI technique with a 1 × 1 mm 2 in-plane resolution that applies motion correction with synthetic image estimation. Motion corrected images were used to generate parametric maps with (T1) and without (T1*) the MOLLI correction. The MOLLI sequence uses a 5 heart-beat (HB) acquisition, 3 HB recovery, 3 HB acquisition scheme with a single shot bSSFP diastolic readout. Images were acquired pre- and 10-25 minutes post- administration of 0.2 mmol/kg gadobenate dimeglumine (Multihance, Bracco Diagnostics, Monroe, NJ). T1 and T1* parametric maps were used to quantify the T1 of tissue and blood, respectively. Two reviewers quantified basal and mid RV free wall, interventricular septal, and lateral LV wall T1 values on T1 parametric maps. RV and LV ECV ranges were calculated assuming normal hematocrit values (women: 0.38-0.46, men: 0.42-0.54). Global ventricular ECV values were compared using the student's t-test. Intra and inter-observer variance was measured by the intraclass correlation coefficient (ICC).

## Results

**Figure 1:**
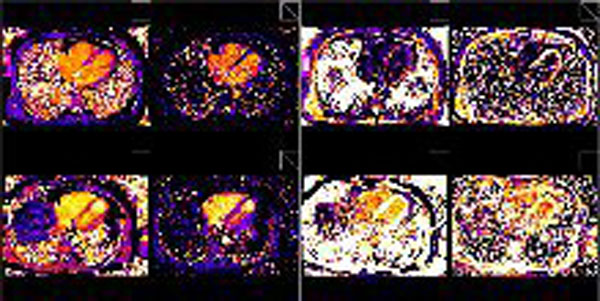
First row: 3T images Second row: 1.5T images Pre contrast T1, pre contrast T1*, post contrast T1, post contrast T1* from left to right for both rows.

Representative T1 and T1* parametric maps are shown in Figure 1. One volunteer (3T) was excluded due to artifact. Table 1 shows RV and LV global ECV ranges by field strength and T1 BP estimation method. Global RV and LV ECV ranges were significantly different at 3T and 1.5T (p < 0.001). A significant influence of field strength was also noted for RV and LV ECV values (p < 0.05). Intraobserver (interobserver) variance for global RV and LV ECV was 0.78 and 0.92 (0.75 and 0.71) at 3T and 0.83 and 0.79 (0.58 and 0.71) at 1.5T, respectively.

**Table 1:** Global RV and LV ECV ranges at 3T and 1.5T as determined using T1 BP values from either the RV or LV on T1 and T1* parametric maps.

	3T				1.5T			
**T1 BP Estimate**	**RVT1**	RVT1* (γ)	**LVT1**	LVT1* (γ)	**RVT1**	RVT1* (γ)	**LVT1**	LVT1* (γ)

RV Global	27-33.5%	26.7-33.1%	24.6-30.8%	26.7-33.5%	24.3-30.8%	24.8-31.5%	24.5-30.8%	24.1-30.6

LV Global	23.5-29.3%	23.3-29.1%	21.4-27%	23.3-29.2%	20.2-26%	20.8-28%	20.1-26%	19.8-25.5%

RV global ECV and LV global ECV calculated using LV values on the T1 parametric map yields unique lower ECV ranges when compared to other blood pool T1 estimation techniques at 3T (p < 0.01). This influence of T1 blood pool estimation on ECV values is not apparent at 1.5T. Interestingly, using T1 parametric maps with T1 BP estimation from the LV at 3T yields RV and LV global ECV values similar to those calculated at 1.5T (p > 0.05). γ: RV global ECV vs LV global ECV, p < 0.001 for each T1 BP estimate comparison. RV T1 = RV blood pool T1 determined on T1 parametric Map, RV T1* = RV blood pool determined on T1*

## Conclusions

This feasibility study demonstrates that HR-MOLLI can quantitate the global RV ECV fraction at both 1.5 and 3T with good intra- and interobserver variance. Our results demonstrated a field-strength influence on RV and LV ECV values, and showed that ECV calculations using blood pool extimates without a look-locker correction are necessary at 3T.

## Funding

Funding pending.

